# Infusion of Phagocytic Macrophages Overexpressing CPT1a Ameliorates Kidney Fibrosis in the UUO Model

**DOI:** 10.3390/cells10071650

**Published:** 2021-06-30

**Authors:** Priscila Calle, Soraya Játiva, Selene Torrico, Angeles Muñoz, Miriam García, Anna Sola, Dolors Serra, Paula Mera, Laura Herrero, Georgina Hotter

**Affiliations:** 1 M2rlab-XCELL, 28010 Madrid, Spain; priscila.calle@iibb.csic.es (P.C.); sjativa@m2rlab.com (S.J.); storrico@m2rlab.com (S.T.); mgarcia@m2rlab.com (M.G.); 2 Department of Experimental Pathology, Instituto de Investigaciones Biomédicas de Barcelona-Consejo Superior de Investigaciones Científicas Institut d’Investigacions Biomèdiques August Pi i Sunyer (IIBB-CSIC-IDIBAPS), 08036 Barcelona, Spain; angles.munoz@iibb.csic.es; 3 Facultat de Medicina, Universitat de Barcelona, 08036 Barcelona, Spain; 4 Department of Experimental Nephrology, Institut d’Investigació Biomèdica de Bellvitge (IDIBELL), L’Hospitalet del Llobregat, 08908 Barcelona, Spain; asola@idibell.cat; 5 Centro de Investigación Biomédica en Red de Fisiopatología de la Obesidad y la Nutrición (CIBEROBN), Instituto de Salud Carlos III, E-28029 Madrid, Spain; dserra@ub.edu (D.S.); paulamera_82@hotmail.com (P.M.); lherrero@ub.edu (L.H.); 6 Department of Biochemistry and Physiology, School of Pharmacy and Food Sciences, Institut de Biomedicina de la Universitat de Barcelona (IBUB), Universitat de Barcelona, E-08028 Barcelona, Spain; 7 CIBER-BBN, Networking Center on Bioengineering, Biomaterials and Nanomedicine, 50018 Zaragoza, Spain

**Keywords:** kidney fibrosis, macrophage, phagocytosis, CPT1a

## Abstract

Phagocytosis is an inherent function of tissue macrophages for the removal of apoptotic cells and cellular debris during acute and chronic injury; however, the dynamics of this event during fibrosis development is unknown. We aim to prove that during the development of kidney fibrosis in the unilateral ureteral obstruction (UUO) model, there are some populations of macrophage with a reduced ability to phagocytose, and whether the infusion of a population of phagocytic macrophages could reduce fibrosis in the murine model UUO. For this purpose, we have identified the macrophage populations during the development of fibrosis and have characterized their phagocytic ability and their expression of CPT1a. Furthermore, we have evaluated the therapeutic effect of macrophages overexpressing CPT1a with high phagocytic skills. We evidenced that the macrophage population which exhibits high phagocytic ability (F4/80low-CD11b) in fibrotic animals decreases during the progression of fibrosis while the macrophage population with lower phagocytic ability (F4/80high-CD11b) in fibrotic conditions, conversely, increases and CPT1a macrophage cell therapy with a strengthening phagocytic ability is associated with a therapeutic effect on kidney fibrosis. We have developed a therapeutic approach to reduce fibrosis in the UUO model by enrichment of the kidney resident macrophage population with a higher proportion of exogenous phagocytic macrophages overexpressing CPT1a.

## 1. Introduction

Kidney fibrosis is a second-line healing program that only occurs if normal kidney repair is impaired or consistently suppressed by ongoing tissue injury and inflammation [1]. Immediately after kidney injury, the innate immune system is activated and orchestrates the recruitment of inflammatory cells from the circulation. Local, resident immune cells, including macrophages, produce chemoattractant that enhance inflammatory responses by recruiting more leukocytes. During tissue injury, changing tissue environments shape the macrophages phenotype towards the anti-inflammatory to provide them with additional functional properties that meet the tissue’s need to address the danger. At the later stage after injury, macrophages contribute to the removal of fibrous tissue, mediating the resolution phase of healing that includes collagen remodeling. However, little is known about how a particular population of macrophages terminates the repair response and becomes pro or anti fibrotic [2]. The knowledge of the agent modulating the phenotype of these macrophages or ex-vivo production of such cells possesses great therapeutic potential.

Nishida et al. [3,4] already suggested the existence of a subpopulation of infiltrating macrophages with an anti-fibrotic role in the recovery phase of obstructive nephropathy in which the angiotensin II type 1 receptor affected macrophage function. Specifically, the group indicates that the number of macrophages and their phagocytic activity is the determinant factor that defines the anti-fibrotic role of macrophages. Zhang et al. [5] revealed that the decrease phagocytosis of pro-fibrotic molecules by macrophages resulted in kidney fibrosis in late-stage UUO. Other studies suggest that macrophages are beneficial in different settings when they may help to remove dead cells and debris through their phagocytic ability [6]. Therefore, it seems clear that macrophages contribute to the removal of fibrous tissue and this fact points to the fundamental role of phagocytosis.

Phagocytosis is an inherent function of tissue macrophages for the removal of apoptotic cells and cellular debris during acute and chronic injury [4]; however, the implications of this event on macrophage function during tissue remodeling and fibrosis are not known.

Overall, we think that despite the macrophage phenotype, the phagocytosis function of macrophages as effector cells able to phagocytize the extracellular matrix and apoptotic cells contributes to fibrosis development. If macrophages are unable to remove cell debris from the extracellular matrix, or are slow to remove it, extracellular matrix accumulation takes place.

Previous studies of our group have evidenced a role of macrophage carnitine palmitoyl transferase (CPT1a) expression in phagocytosis [7]. Furthermore, strategies of CPT1a genetic modification confirmed these findings. In CPT1a knockdown macrophages, we found up-regulated iNOS expression leading to an inflammatory state where phagocytosis was found to be impaired. In accordance, CPT1a overexpression by Crispr activation maintained an improved phagocytosis respect Control Crispr plasmid. Our results revealed that macrophage phagocytosis and inflammatory phenotype are dependent on intracellular lipid accumulation and CPT1a expression. Other authors have found a role of CPT1a expression in kidney fibrosis and recent studies confirm its protective role in epithelial cells [8], but no studies are known about the role of CPT1a on macrophage phagocytosis in kidney fibrosis.

The objective of this work is to know if any population of macrophages during the progression of kidney fibrosis have a reduced phagocytic ability and if increasing the population of phagocytic macrophages in fibrotic kidneys by a new macrophage therapy with highly phagocytic ability could reduce fibrosis development in the experimental model of UUO in mice. For this purpose, we have identified the different macrophage populations during the development of fibrosis and have characterized the phagocytic ability and the expression of CPT1a. Additionally, we have evaluated the capability of a cell therapy with macrophages overexpressing CPT1a, with high phagocytic ability, to ameliorate fibrosis.

## 2. Materials and Methods

### 2.1. Animals

Male CD-1 mice (Crl:CD1 (ICR); Charles River Laboratories Italy Srl, Calco, Italy) of 10–12 weeks old were housed in the Unitat d’Experimentació Animal Facilities of the University of Barcelona at the School of Medicine, with food and water ad libitum in a 12:12 h light/dark cycle. All procedures were previously approved by de Comité Ètic d’Experimentació Animal of the University of Barcelona (CEEA-UB).

### 2.2. Surgical Technique and Experimental Design

Animals were placed in an induction chamber with 5% isoflurane in 1–2 L/min oxygen followed by a maintenance dose at 1.5% with 0.9 L/min oxygen. After confirming a surgical anesthetic plane, ophthalmic ointment was applied, and the surgical field was prepared by removing the hair and disinfecting the skin with a 2% chlorhexidine solution. The animal was then transferred to a sterile field and a suprapelvic transversal incision of 0.5–1 cm was performed. The surrounding external tissue was covered with sterile gauze and the bladder exposed to reveal the left ureter which was ligated with 5/0 silk. Afterwards, the bladder was laid back in place and the incision closed with 4/0 silk. Buprenorphine 0.1 mg/Kg was administered during anesthetic induction and every 6–12 h for a minimum of 72 h. The group of control mice (sham) were operated in the same conditions but without ureter ligation. For the UUO kinetics study, tissues were harvested on day 3, 5 and 7 days (*n* = 4). For the cells therapy groups, one million of macrophages RAW 264.7 (MØ), macrophages RAW 264.7 transfected with adenovirus CPT1AM (AdCPT1AM) and transfected with GPF (AdGPF1), were administered to the mice intravenously through the tail vein on day 3 of UUO and the tissue was then harvested on day 5 after UUO (48 h post-treatment).

### 2.3. Cell Culture

Murine RAW264.7 macrophages, kindly given by Dr. Laura Herrero (Department of Biochemistry and Physiology, University of Barcelona, Spain), were cultured in Dulbecco’s Modified Eagle Medium (DMEM) supplemented with 10% fetal bovine serum (FBS) and 1% penicillin–streptomycin mixture (Gibco, ThermoFisher Scientific, Madrid, Spain). Cells were maintained in a humidified incubator at 37 °C under 5% CO2 and passaged every 2 days when reaching 80% confluence by cell scraping with a slip ratio of 1:3 to 1:6. Cells from passages 9 to 11 were cultured in a 12-well plate at a density of 70,000 cells/well and allowed to grow for 72 h before transduction.

### 2.4. Adenovirus Transduction

We used an adenovirus vector carrying a permanent active mutant form of CPT1a (AdCPT1AM) which is insensitive to its inhibitor’s malonyl-CoA [9] and an adenovirus carrying the green fluorescent protein (AdGFP) as a control. RAW 264.7 macrophages were transduced with AdCPT1AM and AdGFP at a multiplicity of infection (MOI) of 100 for 2 h in serum-free DMEM, and after, replaced with complete medium for an additional 72 h. Adenoviruses aliquots were kindly provided by Dr. Laura Herrero under MTA agreement.

### 2.5. Isolation of Murine Kidney Mononuclear Cells

Kidney mononuclear cells were isolated by enzymatic digestion following a density gradient. Briefly, kidney tissue was decapsulated, mechanically disaggregated into 1–2 mm pieces and enzymatically digested with Collagenase type I (1 mg/mL) for 30 min at 37 °C (Sigma-Aldrich, Madrid, Spain). The cell suspension was then filtered through a 70 µm cell strainer, resuspended in 4 mL DMEM + 20% FBS and centrifugated at 1500 rpm for 5 min at 4 °C. The subsequent pellet was re-suspended in 40% percoll, overlapped on 80% percoll and centrifugated at 400 g for 30 min at room temperature. The obtained fraction was then washed twice and resuspended in facs buffer. Cell counting was preformed using the trypan blue exclusion on a hemocytometer.

### 2.6. Flow Cytometry

Isolated mice kidney cells were pre-incubated with the anti-mouse CD16/32 antibody (Biolegend, San Diego, CA, USA. Cat#:101320) at 1.0 µg/1.06 cells in 100 µL volume for 5 min on ice prior to immunostaining. After, the cells were stained with fluorescence-conjugated surface antibodies for 30 min at 4 °C, washed and analyzed using Becton-Dickinson (BD) FACSCanto II (BD Bioscience, San Jose, CA, USA). Antibodies used for the analysis were: anti-CD45 (Clone: 30-F11) PerCP-Cy5.5 at dilution 1:100 (Biolegend, San Diego, CA, USA. Cat#:103132), anti-F4/80 (Clone: BM8) APC at dilution 1:150 (Biolegend, San Diego, CA, USA. Cat#:123116), CD11b (Clone: M1/70) PE at dilution 1:50 (Biolegend, San Diego, CA, USA. Cat#:101207). Cell sorting was performed with BD FACSAria II (BD Bioscience, San Jose, CA, USA). Facs-sorted macrophages cells (F4/80 high CD11b+ and F4/80low CD11b+) were acquired from a pool of 4 mice per each group (sham and UUO day 5) in order to obtain a pure RNA isolation for qPCR analysis. Data were analyzed using the FlowJo v10 for Windows (FlowJo LLC, Ashland, OR, USA).

### 2.7. Phagocytosis Assay

One vial of pHrodo Green E. coli BioParticles conjugate (Invitrogen, Waltham, MA, USA. Cat#:P35366) was suspended in 2 mL of Live Cell Imaging Solution (LCIS) (Invitrogen, Waltham, MA, USA. Cat#:A14291DJ) at 1 mg/mL, thoroughly vortexed and sonicated following the manufacturer’s instructions. Bioparticles were previously tittered, and the final concentration of 120 μg/mL was our optimal concentration. After the isolation of murine kidney cells, the cells were incubated with pHrodo Green *E. coli* Bio-Particles diluted in LCIS at 120 μg/mL for 90 min at 37 °C. As control, cells were incubated with the same dose of phrodo but at 4 °C. Phagocytosis assay was performed in FACSCanto II. For the phagocytosis assay of the transduced cell, RAW 264.7 transduced cells were incubated with pHrodo Red *E. coli* Bio-Particles (Invitrogen, Waltham, MA, USA. Cat#:P35361) diluted in LCIS at 55 μg/mL for 90 min at 37 °C and the phagocytosis evaluated through fluorescence microscopy and quantify by spectrofluorometer at excitation 560 nm and emission 585 nm.

### 2.8. Histopathological Examination

Transversal cross sections of mice kidney were fixed in 4% paraformaldehyde and embedded in paraffin for histological analysis by hematoxylin-eosin and Sirius red stains. Slides were analyzed by optical microscopy, in a Zeiss Axiophot microscope, to assess fibrosis and were quantified from each non-overlapping cortical field from the cortical region using ImageJ software. For histological analysis, 7 fields per kidney section were evaluated per animal. Pathological evaluation of interstitial fibrosis (IFTA) was assessed following Banff criteria and graded as follows: 0, no damage; 1, Mild interstitial fibrosis and tubular atrophy (<25% of cortical area); 2, Moderate interstitial fibrosis and tubular atrophy (26–50% of cortical area); and 3, Severe interstitial fibrosis and tubular atrophy/loss (>50% of cortical area). Sirius red values were obtained as relative stained area (%) of each field.

### 2.9. Quantitative Real-time Polymerase Chain Reaction (qPCR)

Total RNA was isolated using the RNeasy Mini kit (Qiagen. Hilden, Gerrmany). A total amount of 1μg RNA was reverse transcribed into cDNA using the iScript cDNA synthesis Kit (Bio-Rad, Hercules, CA, USA). qPCR assay was performed on a Bio-Rad CFX96 Touch Real Time PCR detection system with SooAdvanced Universal SYBR Green Supermix (Bio-Rad, Hercules, CA, USA). Levels of mRNA were normalized to those of reference genes GAPDH and 18s rRNA and expressed as fold change respect control or sham. Sequences of specific primers are listed in Table 1.

### 2.10. Statistical Analysis

Data were reported as mean ± SEM of at least three independent experiments. Group means were compared with Student’s t test for two groups or one-way ANOVA followed by Tukey᾿s post-hoc test for multiple comparisons. Values were considered statistically significant if *p* value ≤ 0.05. The analyses were performed in GraphPad Prism version 9.1.0 for Windows (GraphPad Software, San Diego, CA, USA).

## 3. Results

### 3.1. F4/80 Expression in Kidneys Increases According to Fibrosis Degree While CPT1a Expression Decreases

**Figure 1:**
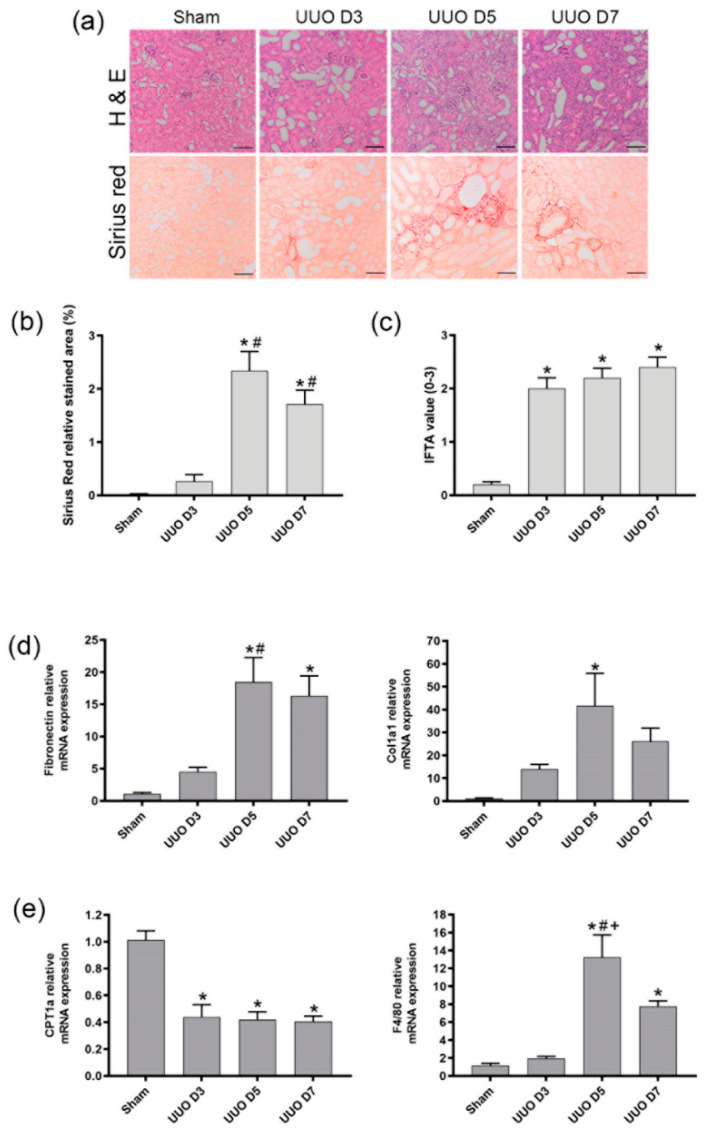
Fibrosis evolution analysis in Unilateral Ureteral Obstruction mice. CD1 mice underwent UUO surgery and kidneys were harvested on days 3, 5 and 7 after surgery. Sham operated mice were used as control. (**a**) Representative images of hematoxylin and eosin (H&E) and Sirius red of kidney sections; objective magnification ×100, scale bar 100 µm. (**b**) Graphical quantification of Sirius red represented as relative stained area (%) and (**c**) H&E using Banff criteria. (**d**–**e**) mRNA levels of (**d**) Fibronectin and Col1a1 and (**e**) F4/80 and CPT1a assessed by qPCR. Levels of mRNA were normalized to those of GAPDH and expressed as fold change. Data presented as mean ±SEM; *n* = 5 per group from 3 independent experiments; * *p* < 0.05 vs. sham. # *p* < 0.05 vs. UUO day 3. + *p* < 0.05 vs. UUO day 7.

Histopathological examination showed a gradual increase in Sirius picric red in relation to the progression of UUO (Figure 1a,b). Severity of IFTA (Figure 1c) increased according to the fibrosis progression. Levels of mRNA fibrosis-related genes, such as col1a1 and fibronectin, during UUO at different time points (day 3, 5, 7) reveal that the main expression peak occurred at day 5 after UUO (Figure 1d), thus indicating that the principal modification occurred during the transition between day 3 and 5 after the obstruction. Interestingly, the enhanced levels of F4/80 mRNA, a macrophage marker, coincided with a sustained down-regulation of CPT1a expression (Figure 1e), indicating a relationship between CPT1a deficiency and macrophage infiltration during fibrosis.

### 3.2. Macrophage Population with Enhanced Phagocytic Ability (F4/80^low^−CD11b+) Decreases during the Progression of Fibrosis While the Population with Reduced Phagocytic Ability (F4/80^high^−CD11b+) Increases

**Figure 2:**
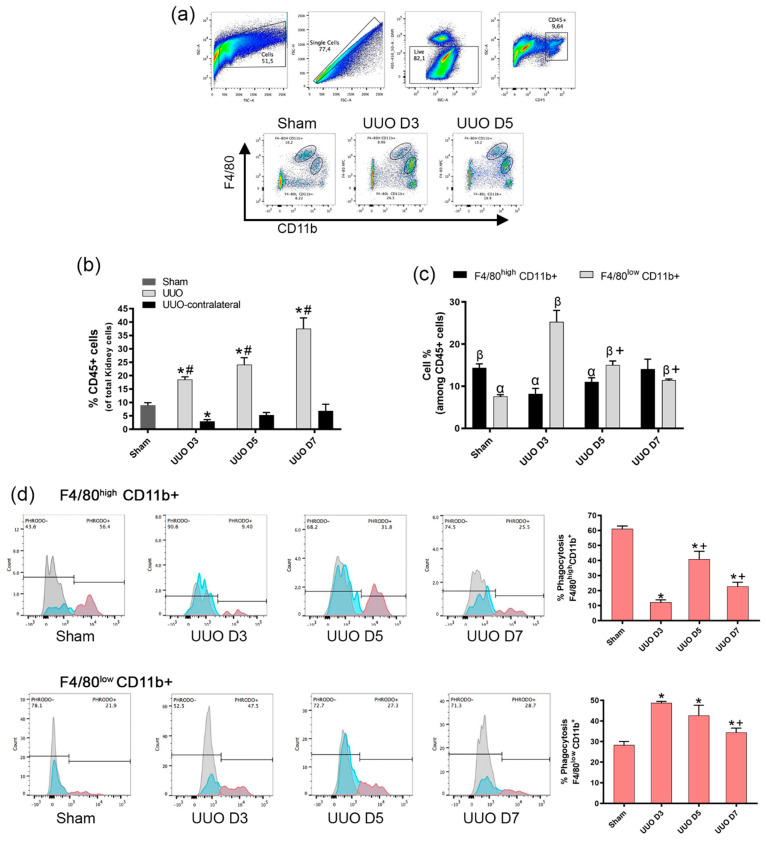
Macrophage phagocytosis analysis during the evolution of fibrosis in the Unilateral Ureteral Obstruction model in mice. Flow cytometry analysis of cells isolated from mice kidney at day 3, 5 and 7 after UUO. Cells isolated from the kidney of sham operated mice were used as control. (**a**) Gating strategy to characterize macrophage population. Live cells (DAPI−) were gated on CD45+ population for analysis of dual expression of surface markers CD11b and F4/80 (oval circles) in the whole kidney at the different time points. (**b**) Percentage of CD45+ cells and macrophage populations (**c**) F4/80^high^−CD11b+ and (**d**) F4/80^low^−CD11b+ at the indicated times. (**d**) Representative analysis of macrophage (defined by gating on CD45+ F4/80^low^−CD11b+ and F4/80^high^−CD11b+) phagocytosis of 120 µg/mL pHrodo green *E. coli* bioparticles for 90 min, at the different time points following UUO. Data presented as mean ± SEM; *n* = 4–6 per group from three independent experiments; *p < 0.05 vs. sham, #*p* < 0.05 vs. UUO-contralateral, +*p* < 0.05 vs. UUO D3. α *p* < 0.05 vs. Sham F4/80^high^CD11b+. β *p* < 0.05 vs. Sham F4/80^low^CD11b+.

To identify the macrophage population in the UUO model of renal fibrosis, we proceed to follow the evolution of UUO in order to isolate the macrophages on different days and characterize them by flow cytometry, Once the enzymatic digestion and gradient centrifugation of each kidney was carried out, the isolated cells were labelled with anti-CD45, anti-CD11b, anti-F4/80 antibodies to perform the gating strategy for detecting macrophages (Figure 2a) and later incubated with pHrodo E. coli bioparticles at 37 °C and at 4 °C to evaluate macrophage phagocytosis at the different time points of fibrosis development.

The presence of CD45+ cells increased proportionally with the days of ureteral obstruction and were heightened when compared to the sham kidney and the un-obstructed contralateral kidney of UUO mice, displaying the recruitment of CD45+ leukocytes throughout fibrosis (Figure 2b). To identify macrophages, the CD45+ cells were further analyzed for the expression of the cell-surface markers CD11b and F4/80. We detected two subtypes of macrophages depending on the intensity expression of F4/80 (specific murine macrophage marker). In sham operated mice, the CD11b+F4/80^high^ fraction was significantly higher than the CD11b+F4/80^low^ fraction, but the latter increased following day 3 of UUO surgery, while the former trended to be reduced (Figure 2c). The presence of both populations exhibited opposite behavior at the different time points of UUO, as F4/80^high^−CD11b+ increased significantly during fibrosis progression with respect to day 3 after UUO, while F4/80^low^−CD11b+ cells, on the contrary, decreased significantly (Figure 2c).

Phagocytosis analysis by pHrodo in the characterized populations indicated that the phagocytic ability of the CD11b+F4/80^high^ cells was significantly higher than the CD11b+F4/80l^ow^ cells in the sham operated mice (Figure 2d). Nevertheless, after UUO, the CD11b+F4/80^high^ population exhibited less phagocytosis than the CD11b+F4/80^low^.

Thus, tissue macrophages display a different ability to phagocytize during the progression of fibrosis, altogether indicating that in fibrotic kidneys, there is a decrease in the net phagocytosis of macrophages by two simultaneous mechanics: 1) a decrease in the population of F4/80^low^−CD11b+ that showed a higher phagocytosis in fibrotic conditions, and an increase in the F4/80^high^−CD11b+ population which showed s lower phagocytic function in fibrotic conditions.

### 3.3. Gene Expression Profile of F4/80^high^−CD11b+ and F4/80^low^−CD11b+ Macrophages Isolated from Obstructed Kidney

The different macrophage populations isolated at day 5 following UUO were sorted and mRNA levels of CPT1a, PPAR-α, iNOS, IL-10, NLRP3 and IL-1β were evaluated.

**Figure 3:**
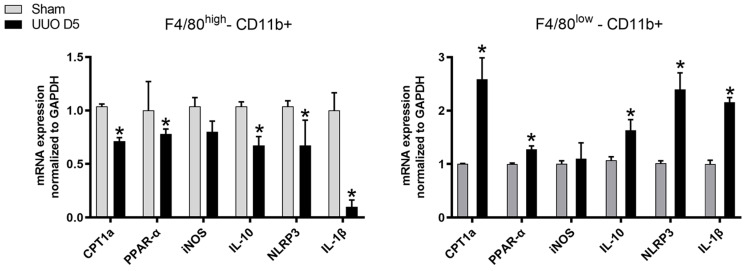
Gene expression profile of macrophages isolated from kidneys. Cells isolated from kidney tissue of sham operated and UUO-induced fibrotic mice at day 5 after obstruction characterized by flow cytometry. mRNA levels of CPT1a, PPAR-α, IL-10, iNOS, NLRP3 and IL-1β from sorted macrophages measured by qPCR. Levels of mRNA were normalized to those of GAPDH and expressed as fold change. Data presented as mean ± SEM; *n* = 4 per group from three independent experiments; * *p* < 0.05 vs. sham.

As shown in Figure 3, the F4/80^high^−CD11b+ population which exhibited a decline in phagocytosis expressed a significant down-regulation of CPT1a, PPAR-α, IL-10, NLRP3 and IL-1β whereas the decreasing population of F4/80^low^−CD11b which presented a heightened phagocytosis revealed a significant overexpression of CPT1a, PPAR-α, IL-10, NLRP3 and IL-1β.

### 3.4. Macrophage Gene Expression Profile and Phagocytosis Assay of Macrophages Overexpressing CPT1a before Infusion

Since impaired macrophage phagocytosis connects with a deficient CPT1a expression in UUO, macrophages Raw 264.7 were genetically modified to overexpress a permanently active mutant form of CPT1a (CPT1AM) to serve as a cell therapy in UUO. To confirm the efficiency of transduction, CPT1a mRNA levels were determined by qPCR. In addition, the levels of the inflammasome NLRP3 and the inflammatory marker iNOS were measured to establish the effects of adenovirus infection.

**Figure 4:**
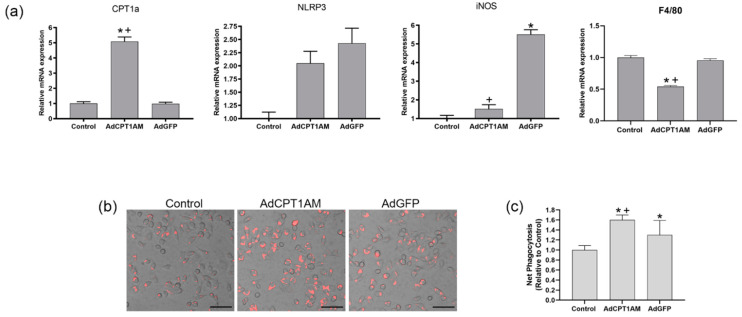
Phagocytic ability and mRNA analysis of CPT1AM-expressing Raw 264.7 macrophages. Murine macrophage Raw 264.7 was transduced with adenovirus carrying CPT1AM (AdCPT1AM) and GPF (AdGFP). Untransfected cells served as control. (**a**) mRNA expression determined by qPCR of CPT1a, NLRP3, iNOS and F4/80. (**b**) Representative images of pHrodo uptake captured by fluorescence microscopy. Objective magnification ×400, scale bar 50 µm. (**c**) Net Phagocytosis of 55 µg/mL pHrodo Red E. coli bioparticles conjugate for 90 min measured by spectrofluorometer. Data presented as mean ± SEM; *n* = 6 of three independent experiments; * *p* < 0.05 vs. control. + *p* < 0.05 vs. AdGFP.

As shown in Figure 4A, CPT1a mRNA levels on cells transduced by adenovirus CPT1AM (AdCPT1AM) produced a significant increased (5-fold change) compared with control cells and macrophages transfected with adenovirus GFP (AdGFP). Although iNOS mRNA levels overexpressed in AdGFP cells, in AdCPT1a cells, the levels were not statistically significant and maintained the same levels as the control. On the other hand, the risen levels of mRNA NLRP3 in both adenoviruses AdCPT1AM did not reach statistical significance. In addition, an effect on F4/80 expression in AdCPT1AM cells was observed. Thus, macrophages that overexpress CPT1a do not have an inflammatory profile. As shown in Figure 4B, the phagocytic ability of CPT1a overexpressing macrophages was augmented with respect to macrophages AdGFP.

The effect of CPT1a on the phagocytic capacity of peritoneal macrophages is shown in Figure S1. We compared the phagocytic ability of peritoneal macrophages CPT1a silenced with peritoneal macrophages silenced with β-gal. The phagocytic ability was evaluated by the ability to internalized zymosan particles and CPT1a mRNA levels analyzed by q-PCR. Results reflect a decrease of phagocytosis when CPT1a was silenced in peritoneal macrophages (Figure S1).

### 3.5. Cell Therapy with a Stable Population of Macrophages Overexpressing CPT1a, with High Phagocytic Ability, Reduced Fibrosis in the UUO Model

**Figure 5:**
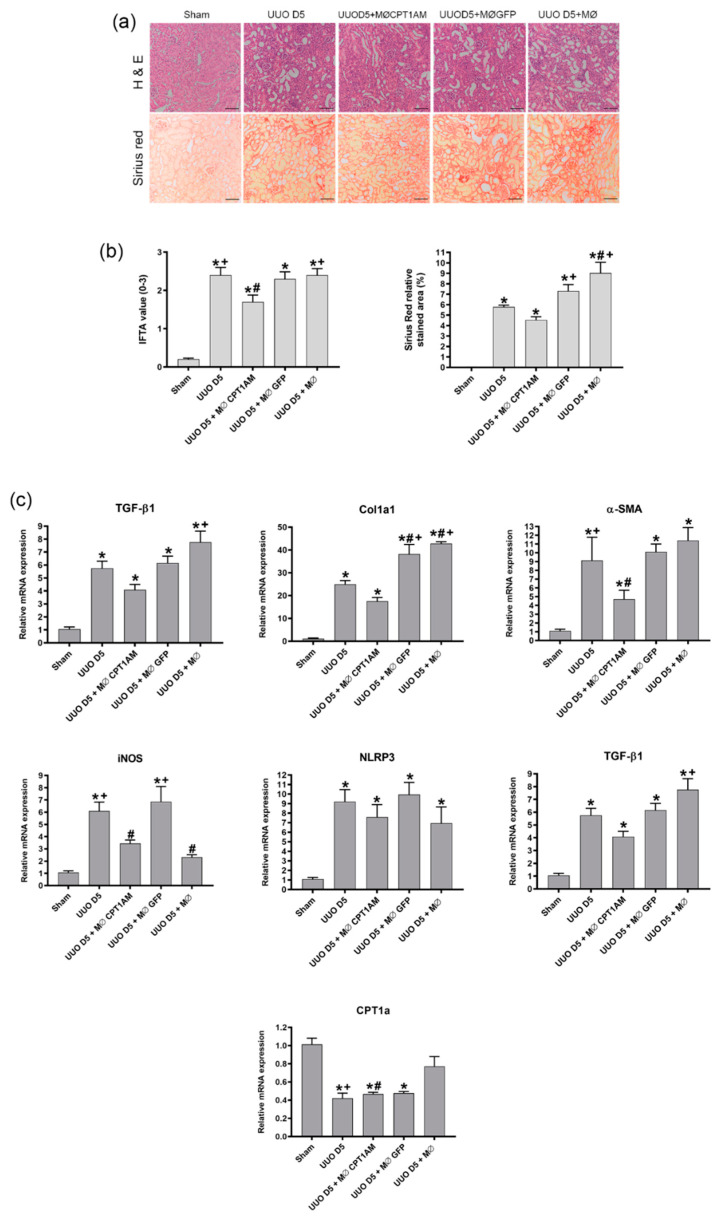
Effect of CPT1a macrophages in UUO-induced fibrosis mice. Evaluation of UUO operated mice with and without intravenous infusion of cell therapy on day 3 after UUO. Kidneys were harvested 48 h after cell therapy at day 5 following UUO. (**a**) Representative images of hematoxylin and eosin (H&E) and Sirius red. Objective magnification ×100, scale bar 100 µm (**b**) Graphical quantification of H&E and Sirius red from each group using Banff criteria and relative stained area (%), respectively. (**c**) mRNA levels of Col1a1, fibronectin, α-SMA, NRLP3, TGF-β1, iNOS and CPT1a of whole kidney tissue examined by qPCR. Data presented as mean ± SEM; *n* = 4 per group from 3 independent experiments; * *p* < 0.05 vs. sham. # *p* < 0.05 vs. UUO D5. + *p* < 0.05 vs. UUO D5 + MØCPT1AM.

As shown in Figure 5a, histological analysis of the different groups indicated that mice that underwent UUO and were infused with macrophages overexpression CPT1a (UUO D5 + MØCPT1AM) showed a reduced IFTA and a reduced intensity of Sirius red (Figure 5b), thus reflecting the protection from fibrosis development.

As shown in Figure 5c, cell treatment with MØCPT1AM was associated with a significant reduction of the fibrotic marker’s fibronectin, col1a1, α-SMA and TGF-β1 as compared to the therapies of the vehicle group of macrophages (UUO D5 + MØ) and the transduction control group (UUO D5 + MØGFP) as well as with the untreated animals that experienced UUO for 5 days (UUO D5). Likewise, NLRP3, iNOS were also diminished in response to MØ CPT1AM cell therapy, although the NLRP3 decrease did not reach significance with respect to the mice UUO D5. Nevertheless, CPT1a expression continued downregulated with respect to sham mice in all the groups.

**Figure 6:**
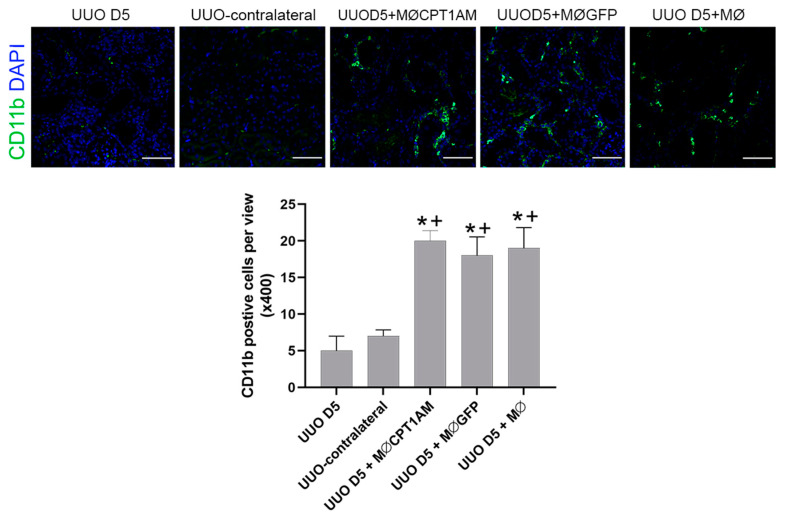
CD11b positive cells in Kidney tissue of UUO-induced fibrosis mice with or without macrophage cell therapy. Immunofluorescence of kidney section with the antibody against CD11b (green); nuclei were stained with DAPI (blue) in UUO operated mice that received, at day 3 of UUO, an intravenous injection of macrophages transduced with either CPT1a or GFP, and macrophage without transduction. Kidneys were harvested 48 h after cell therapy at day 5 following UUO. Vehicle group received an intravenous injection of PBS (UUO D5) and contralateral kidney of UUO mice served as control. Objective magnification ×400, scale bar 50 µm. * *p* < 0.05 vs. UUO day 5. + *p* < 0.05 vs. UUO-contralateral.

Immunofluorescent staining of CD11b in tissue after cell therapy with the different treated macrophages of the study is showed in Figure 6. As observed, kidney from UUO mice treated with MØCPT1AM, MØGFP or MØ showed CD11b positive cells in kidney tissue with respect to the vehicle group (UUO D5 +) and the non-obstructed contralateral kidney of the UUO operated mice (UUO-contralateral) indicating that macrophages reached and remained in damaged kidney tissue after intravenous cell therapy administration.

In addition, the effect of CPT1a inhibition on promoting fibrosis in the UUO model is shown in the Figure S2. Mice that underwent UUO surgery were administered the synthetic compound C75 which inhibits CPT1 activity. Results showed a decreased CPT1a expression and an increase in the fibrotic markers α-SMA and Col1a1 while TGF-β1 decreased.

## 4. Discussion

Among the main macrophage phenotypes, it seems that M1 (inflammatory macrophages) and M2 (regenerative macrophages) are involved in the fibrotic process, but the involvement is controversial and, in some studies, depends on the fibrosis stage after injury. It has been found that elimination of M2 macrophages at the advanced stage of fibrosis in UUO animals mitigated the formation of renal fibrosis, but elimination of M1 macrophages in the early UUO stage did not modify the fibrotic features [6]. In the same sense, other authors indicated that it was M2 macrophage depletion, rather than M1, that specifically inhibited the mesenchymal epithelial transition and, therefore, renal fibrosis [10]. On the contrary, others found that the increase in the proportion of M2 macrophages plays an important beneficial role in the resolution of renal fibrosis, being the most characteristic feature of the early recovery phase after UUO [11]. Recent studies in our group indicate that intravenous administration of a stable population of M2 macrophage reduces the impact of the fibrosis [12].

Macrophage dynamics during the different phases of CKD progression are not fully known, and assessment of the predominant macrophage phenotype may be relevant in terms of defining the type of therapy [13].

Anders and Ryu [14] have proposed to classify tissue macrophages according to their predominant roles in different phases of kidney disease: pro-inflammatory, anti-inflammatory, profibrotic and fibrolytic macrophages. However, there is a lack of information regarding macrophage types and their dynamics, plasticity, and function in human CKD. Macrophages represent a spectrum of activated phenotypes rather than discrete stable subpopulations. Therefore, better strategies, based in the macrophage function and phenotype, should be studied to induce truly regenerative and reparative macrophages therapies.

Belliere et al. described a switch of macrophage phenotype from M1 to M2 during disease progression in a mouse model of rhabdomyolysis-induced AKI [15], whereby F4/80^low^CD11b^high^ M1 kidney macrophages were dominant 2 days after the induction of rhabdomyolysis, whereas F4/80^high^CD11b+ M2 cells were dominant by day 8. Importantly, analysis at the single cell level indicated the existence of considerable phenotypic heterogeneity, with some macrophages expressing both M1 and M2 markers. Likewise, an M1 to M2 transition was also evident in a UUO model of renal fibrosis, which was promoted by the actions of a tissue type plasminogen activator [16].

Several publications strongly suggest that the expression of F4/80+CD11b+ cells in the obstructed kidneys starts going up after at least 4 days of UUO [17,18,19]. Our results are in line with these results, indicating that under control conditions (sham mice), the F4/80^high^CD11b+ fraction was significantly larger than the F4/80^low^CD11b+ fraction (Figure 4b), but the latter was increased at day 3 after UUO, while the former was reduced on day 3. Nonetheless, during the UUO progress, F4/80^high^CD11b+ increases as other authors suggest, while the F4/80^low^CD11b+ decreases during the progression of fibrosis.

Our study has identified two different types of macrophages which exhibit a differential phenotype, being the F4/80^low^CD11b+ population with high phagocytosis predominant at the initial phases of injury, while the F4/80^high^CD11b+ with low phagocytosis was dominant at the later phases of UUO development. Moreover, there is a net decrease in phagocytosis, since the population with higher phagocytic ability decreases with respect to day 3 following ureter obstruction, while the population with low phagocytic ability increased as shown in Figure 2. Further, when cells were isolated from fibrotic kidneys, the F4/80^high^ population, characterized by low phagocytosis, expressed a decrease of PPAR-α expression and its target gene CPT1a as well as IL-10, NLRP3, IL-1β compared to the F4/80^low^ population of macrophages which presented elevated mRNA levels.

Some authors previously [17] indicated that the CD11b+F4/80^low^ has shown a phenotype profile of M1-type activation, while CD11b+F4/80^high^ shows a phenotype indicative of M2-type activation.

Our results indicate that the phenotype of CD11b+F4/80^low^ could be like M1 since an increase in NLRP3 and IL-1β could be observed, but the fact that IL-10 also increased indicates a mixed phenotype. In contrast, CD11b+F4/80^high^ has a trend towards M2 phenotype, since NLRP3 and IL-1β are decreased, but the diminished levels of IL-10 point to a mixed phenotype again. However, our intention was to determine whether these two populations differ in CPT1a expression, which has been confirmed in the present study.

Other studies [20] previously confirmed marked changes in the proportion and gene expression of at least 12 myeloid cell subsets involved in kidney injury and repair in a reversible UUO model. In addition, they described a novel Mmp121 macrophage subset that acts during repair. The Mmp121 cluster–expressed genes are implicated in efferocytosis and lipid transport, suggesting that they may be involved in the clearance of apoptotic cells, thus pointing again to a potential role of macrophage phagocytosis in kidney repair.

The association of defective phagocytic ability of mononuclear cells and exaggerated inflammatory and fibrotic responses has been proposed earlier [21] and the F4/80^high^ population, which has been characterized as embryo-derived macrophages, are known to exhibit a higher phagocytic ability than F4/80^low^ bone marrow-derived macrophages (BMDM) [22]. Our results agree with these authors, since in sham conditions, the F4/80^low^ CD11b+ cells are more phagocytic that F4/80^high^ CD11+, but in fibrotic conditions, there is a shift between populations as F4/80^low^ CD11b+ become less phagocytic than the F4/80^high^ CD11b+.

One potential explanation could be that after UUO, the F4/80^high^ CD11b+ population exhibits a decrease in the expression of CPT1a that leads to a decrease in phagocytosis since a direct relationship was previously observed between CPT1a as inductor of phagocytosis [7]. One could speculate that in fibrotic conditions, the higher phagocytic population, the F4/80^high^ CD11b+population, is able to phagocytose detritus and to be converted in foam cells, thus leading to a downregulation of CPT1a that in turns reduces the phagocytosis of this population during fibrosis. On the other hand, the F4/80^high^ CD11b+ cells have been previously described as embryo derived macrophages with high glycolysis [22] that could also potentially result in a decrease in CPT1a expression as observed in our experiments. Nevertheless, the present study has not addressed these issues.

These observations support the importance of macrophage dynamics during fibrosis development. and suggest a potential therapeutic approach to reduce fibrosis in the UUO model by enrichment of the kidney resident macrophage population with a higher proportion of exogenous phagocytic macrophages.

We have tested the effect of a therapy with a stable population of macrophages which exhibit high phagocytosis ability.

As indicated in Figure 4, macrophages overexpressing CPT1a have a high phagocytic ability. This has been observed in previous studies [7] that revealed that CPT1a overexpression decreases lipid content and stimulates phagocytosis while CPT1a knockdown promotes an increase in lipid accumulation and in the pro-inflammatory marker iNOS associated with an impaired phagocytosis and down-regulation of NLRP3. Other authors have observed that a NOX4 dependent inactivation of CPT1a leads to a reduction in NLRP3 activation in macrophages [23]. This study indicates that CPT1a overexpressing macrophages show an increase in NLRP3 and iNOS expression but without statistical significance, suggesting that this level of CPT1a overexpression does not lead to an inflammatory profile. In addition, as observed in Figure S2, the C75 compound, which is known to inhibit CPT1a activity, induced an increase in the fibrotic markers although a reduction in TGF-β1 was observed. Therefore, it could be said that CPT1a expression is related to the development of fibrosis although TGF-β1 is decreased.

In addition, the phagocytic ability of macrophages overexpressing CPT1a is superior; hence, using these cells as a therapy could reestablish the reduced macrophage phagocytosis observed in kidney fibrosis.

These observations suggest that the expression of CPT1a in macrophages could counteract the increase in the low phagocytic (F4/80^high^−CD11b) population and that the increase in phagocytic macrophages results in an amelioration of fibrosis.

Our results indicate that cell therapy with macrophages overexpressing CPT1a with high phagocytic ability applied to the UUO model reduces the expression of the fibrotic markers, fibronectin, α-SMA, Col1a1 and TGF-β1, indicating the potential of our therapy to revert fibrosis development. Previous studies in the UUO model have demonstrated that macrophage therapy with a stable population of M2 reduced the impact of fibrosis in front of other macrophage therapies which do not exhibited a stable phenotype [12]: thus, indicating that if macrophages maintain a stable M2 phenotype, fibrosis could be overcome.

## 5. Conclusions

In conclusion, we have identified the dynamics of two populations of macrophages during fibrosis development, with different phagocytic ability. We demonstrated firstly that during fibrosis development, there is a net decrease in macrophage phagocytosis since the macrophage population with high phagocytic ability in fibrotic conditions (F4/80^low^−CD11b+) decreases during the progression of fibrosis while the macrophage population with low phagocytic ability (F4/80^high^−CD11b+) increases during the progression of fibrosis; and secondly, that macrophage-CPT1a cell therapy with a high phagocytic ability is associated with a therapeutic effect on kidney fibrosis.

## Figures and Tables

**Table 1:** Primers sequences used for qPCR.

Gene	Forward Primer	Reverse Primer
*GAPDH*	TGAAGCAGGCATCTGAGGG	CGAAGGTGGAAGAGTGGGAG
*18s rRNA*	CCTGCGGCTTAATTTGACTC	GACAAATCGCTCCACCAACT
*CPT1a*	TTTGAATCGGCTCCTAATGG	CCCAAGTATCCACAGGGTCA
*TGF-β1*	ATTCCTGGCGTTACCTTGG	CCTGTATTCCGTCTCCTTGG
*iNOS*	AGGGAATCTTGGAGCGAGTT	GCAGCCTCTTGTCTTTGACC
*Col1a1*	CGATGGATTCCCGTTCGAGT	GCTACGCTGTTCTTGCAGTG
*Fibronectin*	AGACTGCAGTGACCACCATTC	AATGTGTCCTTGAGAGCATAGAC
*F4/80*	GCCCAGGAGTGGAATGTCAA	GCAGACTGAGTTAGGACCACA
*Il-10*	CATGGGTCTTGGGAAGAGAA	AACTGGCCACAGTTTTCAGG
*α-SMA*	TCCAGCCATCTTTCATTGGGA	CCCCTGACAGGACGTTGTTA
*IL-1β*	TGCCACCTTTTGACAGTGATG	ATGTGCTGCTGCGAGATTTG
*PPAR-α*	AAAGAGGCAGAGGTCCGATT	AGCAAGGTGACTTGGTCGTT
*NLRP3*	Assay ID: qMmuCID0010647	PrimePCR™ SYBR® Green Assay (Bio-Rad)

## Data Availability

The data that support the findings of this study are available from the corresponding author upon reasonable request.

## Supplementary Materials

The following are available online at https://www.mdpi.com/article/10.3390/cells10071650/s1, Figure S1: Effects of CPT1a-2 knockdown on peritoneal macrophages phagocytosis, Figure S2: Effects of CPT1a inhibition by C75 in UUO induced fibrosis mice.
