# Supplementary material for: Infusion of Phagocytic Macrophages Overexpressing CPT1a Ameliorates Kidney Fibrosis in the UUO Model
Source: Cells. 2021 Jun 30;10(7):1650. doi: 10.3390/cells10071650 (PMC8304788; doi:10.3390/cells10071650)
Supplement: Supplementary file 1 [file cells-10-01650-s001.zip › cells-1223877-supplementary.pdf]

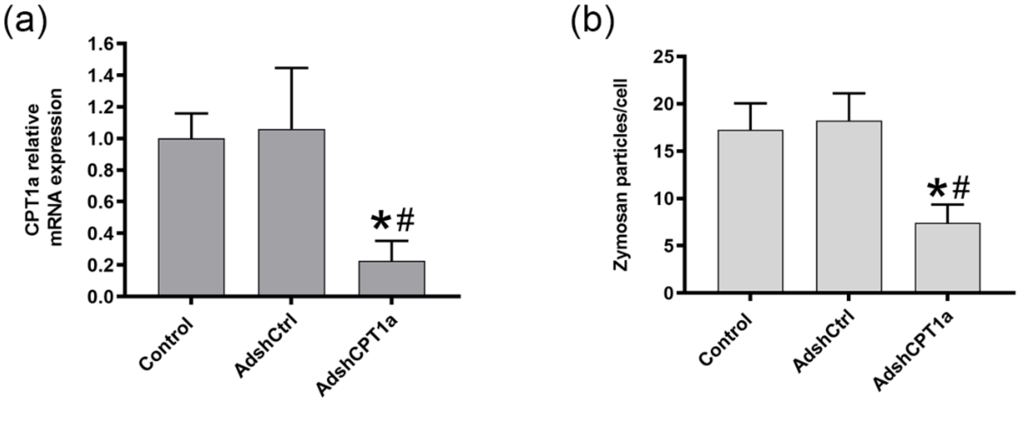

Figure S1. Effects of CPT1a-2 knockdown on peritoneal macrophages phagocytosis. Mouse peritoneal macrophages obtained after intraperitoneal injection of thioglycollate medium 3%, were plated in RPMI medium supplemented with 10% fetal bovine serum (FBS) and 1% penicillin/streptomycin. Cells were maintained in a humidified incubator at 37 °C under 5% CO2. Macrophages were subsequently isolated by adherence after 24h. Macrophages were transduced with adenovirus vector carrying a short hairpin targeting Cpt1a (AdshCPT1a) at MOI 150 and a non-targer hairpin (AdshCtrl) at MOI 100, in antibiotic free RPMI supplemented with 2% FBS for 24 h. a) CPT1a expression was determined by qPCR and b) phagocitosis assay was performed. One vial of Zymosan A BioParticules (Z-23373 Molecular Probes) were tittered to a final concentration of 100 particles per cells for the assay for 60 min at 37 °C, following the manufacturer instructions. The influence of CPT1a silencing on phagocytosis were evaluated. Data presented as means ± SEM, *p ≤ 0.05 vs. Control. #p ≤ 0.05 vs. AdshCtrl.

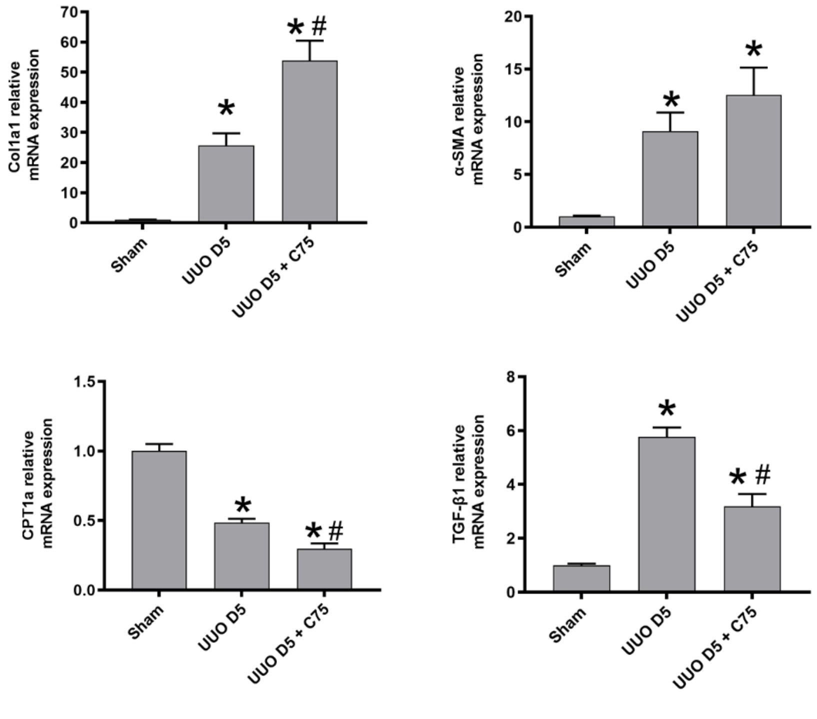

Figure S2. Effects of CPT1a inhibition by C75 in UUO induced fibrosis mice. Evaluation of UUO operated mice with and without intraperitoneal injection of 15mg/kg of C75 on day 3 after UUO. Kidney were harvest 5 days followed UUO (48h after injection). mRNA levels of Col1a1, α-SMA, TGF-β1 and CPT1a of whole kidney tissue assessed by qPCR. Data presented as mean ± SEM; *p < 0.05 vs. sham. # p < 0.05 vs. UUO day 5.
